# Therapeutic development targeting host heparan sulfate proteoglycan in SARS-CoV-2 infection

**DOI:** 10.3389/fmed.2024.1364657

**Published:** 2024-03-27

**Authors:** Qi Zhang, Ivan Pavlinov, Yihong Ye, Wei Zheng

**Affiliations:** ^1^Therapeutic Development Branch, National Center for Advancing Translational Sciences, National Institutes of Health, Bethesda, MD, United States; ^2^Laboratory of Molecular Biology, National Institute of Diabetes, Digestive, and Kidney Diseases, National Institutes of Health, Bethesda, MD, United States

**Keywords:** HSPG, COVID-19, viral entry, drug development, viral entry and infection

## Abstract

The global pandemic caused by the severe acute respiratory syndrome coronavirus 2 (SARS-CoV-2) has led to an urgent need for effective therapeutic options. SARS-CoV-2 is a novel coronavirus responsible for the COVID-19 pandemic that has resulted in significant morbidity and mortality worldwide. The virus is known to enter host cells by binding to the angiotensin-converting enzyme 2 (ACE2) receptor, and emerging evidence suggests that heparan sulfate proteoglycans (HSPGs) play a crucial role in facilitating this process. HSPGs are abundant cell surface proteoglycan present in many tissues, including the lung, and have been shown to interact directly with the spike protein of SARS-CoV-2. This review aims to summarize the current understanding of the role of HSPGs in SARS-CoV-2 infection and the potential of developing new therapies targeting HSPGs.

## Introduction

COVID-19 is a highly infectious disease caused by SARS-CoV-2, a novel coronavirus emerged in Wuhan, China, in late 2019. The virus enters human bodies primarily via the respiratory system, leading to a range of symptoms, from mild flu-like symptoms to severe acute respiratory syndrome (SARS) and multi-organ failure. The rapid spread of the virus has led to a global pandemic, with over 757 million confirmed cases and over 6.8 million deaths reported as of February 2023 according to the World Health Organization’s weekly update. Despite extensive efforts to develop effective therapies and vaccines, the emergence of new viral variants has posed a continued challenge to the public health (1).

The spike protein of SARS-CoV-2 binds to ACE2 receptor that mediates viral entry via either the endocytosis or the plasma membrane fusion routes. The spike protein is a type I membrane protein consisting of two proteolytically cleaved fragments, S1 and S2, which are the products of the host Furin protease. The entry route is largely influenced by the availability of additional cellular proteases on target cells, which cleave the S2 fragment to activate the fusion reaction. TMPRSS2 and cathepsin L are the two major proteases involved in spike protein activation (2). The interaction of the Spike with ACE2 on the cell surface can be influenced by the cell surface heparan sulfate proteoglycan (HSPG), which serves as a coreceptor to facilitate viral entry (3). Additionally, HSPG appears to modulate the oligomerization state of ACE2 to promote Spike induced membrane fusion (4).

Following ACE2 binding, TMPRSS2-mediated cleavage of the S2 fragment occurs on the plasma membrane, exposing the fusogenic peptide in the spike protein to drive the fusion of the viral membrane with the host plasma membrane resulting release of the viral genome to the cytoplasm (5). Alternatively, Cathepsin L cleaves the S2 subunit within the late endosome and lysosome, following viral entry via ACE2-mediated endocytosis, lysosome membrane fusion, and subsequent release of the viral RNA into the cytoplasm (2, 5, 6). Furin, a host cell protease, plays a pivotal role in the activation of the SARS-CoV-2 spike (S) protein by cleaving it at the S1/S2 site mainly in the Golgi. This proteolytic cleavage is critical for the S protein to undergo the necessary conformational changes for viral entry into host cells (7). Since Furin was also detected in human serum and a prognostic role of serum Furin was suggested (8), extracellular Furin might contribute to Spike processing, and therefore, the cell entry of SARS-CoV2. Additionally, Furin was found to constantly recycle between the cell surface and endosomes with a small fraction detected on the cell surface (9), the cell surface-localized Furin was suggested to facilitate viral entry by cleaving the full-length Spike proteins that escape S1/S2 cleavage during viral biogenesis (10). The precise function of extracellular Furin in SARS-CoV-2 entry and whether HSPG regulates this process remains to be elucidated.

Because the entry of SARS-CoV-2 is primarily mediated by the binding of the spike protein to ACE2, agents inhibiting ACE2-spike protein binding either directly or indirectly (such as targeting cell surface HSPG) can block viral entry. Several monoclonal Spike antibodies that inhibit the spike-ACE2 interactions showed very good therapeutic efficacy and had been approved for treatment of SARS-CoV-2 infection (11). However, due to the rapid evolving of SARS-CoV-2 variants, all these approved monoclonal antibodies lost efficacy to new SARS-CoV-2 variants. Because the host factors are much less affected by viral mutations, small molecule inhibitors that directly target HSPGs have potential for the next generation of drugs against SARS-CoV-2 infection.

## Evolving of SARS-CoV-2 variants

**Figure 1:**
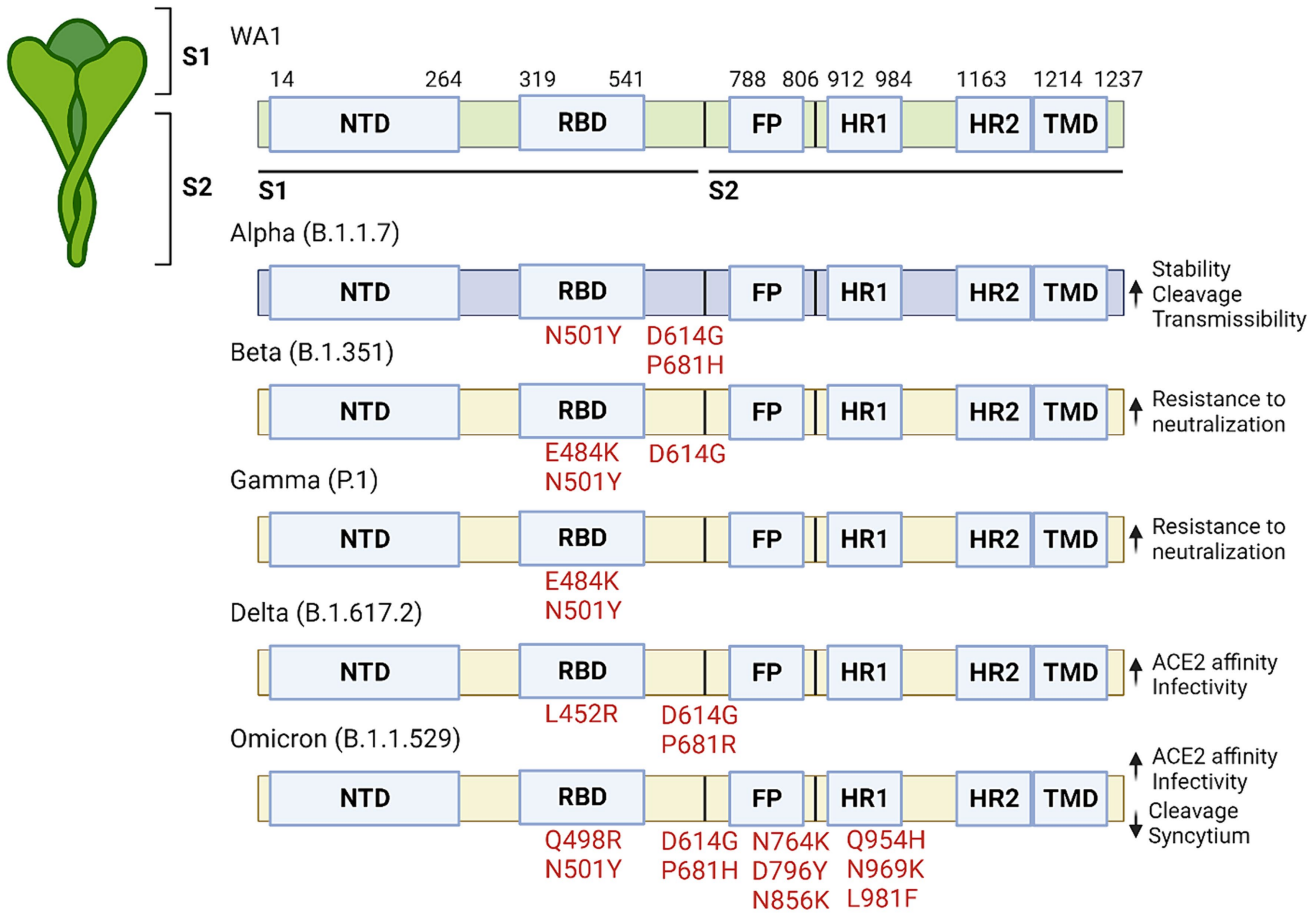
Significant mutations that are present in variants of concern. The SARS-CoV-2 protein is made up of two subunits (S1 and S2) which are further subdivided into the NTD, RBD, FP, HR1, HR2 and TMD domains. Significant mutations are listed by their relative location within the protein along with the corresponding affect to the right.

There have been five major variants of concern (VOC) that have been extensively studied (12–16). These include the Alpha variant (B.1.1.7), Beta variant (B.1.351), Gamma variant (P.1), Delta variant (B.1.617.2), and Omicron variant (B.1.1.529). Each variant has specific characteristics that affect viral transmissibility, virulence, and immune evasion (Figure 1).

The Alpha variant, first identified in the United Kingdom in December 2020, has mutations in the spike protein (N501Y, D614G and P681H) which increased spike protein stability and furin cleavage, leading to increased affinity to human cells (17–19). This heightened contagiousness led to its rapid global spread. The Beta variant, discovered in South Africa in December 2020, carries mutations that may help it evade certain immune responses while spreading more rapidly. In addition to having the N501Y and D614G mutation it also carries aK417N mutations which further enhances the binding between spike and ACE2, whereas the E484K mutation is associated with increased resistance to neutralizing antibodies (17, 18, 20, 21). The Gamma variant, detected in Brazil in January 2021, has some of the same mutations as those in the Alpha and Beta spike proteins, which allow it to attach more easily to human cells and also evade immunity more effectively (22–26). The Delta variant, which emerged in India in December 2020, was the dominant strain in many countries before the emergence of the Omicron variant. Vaccines appear to be less effective against the Delta variant than the Alpha strain, although they are still effective at preventing hospitalization after two doses, likely due to the L452R mutation in the RBD which lowers antibody affinity for the spike protein (27). The Omicron variant was first identified in South Africa in November 2021, and is characterized by numerous mutations in the spike protein. These mutations contribute to increased transmissibility and immune evasion, making it the dominant strain worldwide. Compared to the original SARS-CoV-2 virus and the Delta variant, the Omicron variant spreads more easily, but appears to cause milder symptoms, likely due to its preference for entering cells via the endocytosis pathway (28). The variant possesses six unique mutations on the S2 protein (Q498R, N764K, D796Y, N856K, Q954H, N969K, and L981F) that have not been identified in previous VOC. Although it replicates faster in primary cultures of human nasal epithelial cells, it amplifies at a lower replication rate in lung and gut cell lines, likely due to further increases in ACE2 affinity caused by the dual Q498R + N501Y mutation (29). These findings suggest that Omicron tends to infect the upper respiratory tract may contribute to its improved prognosis Furthermore, compared to the Delta variant, the Omicron strain has reduced ability to cause syncytium formation and shows suboptimal S1/S2 cleavage, potentially limiting its virulence (30, 31).

Although the different SARS-CoV-2 variants differ in transmissibility and resistance to existing immunity, the clinical symptoms of SARS-CoV-2 infection are generally similar across all major variants, which include fever, dry cough, fatigue, shortness of breath, muscle aches, headache, loss of taste or smell, sore throat, congestion, nausea, vomiting, and diarrhea. However, the severity of the symptoms can vary significantly, with some infected individuals being asymptomatic. The Alpha, Delta, and Omicron variants are more transmissible than the original strain, potentially causing different patterns of disease spread. The Beta and Gamma variants show greater potential for immune evasion, which may reduce the effectiveness of some vaccines or natural immunity from previous infections. Due to the propensity of the virus to rapidly mutate and acquire these types of mutation it is important that nonviral targets are investigated for their potential to block viral infection or replication.

## HSPG facilitates SARS-CoV-2 infection

Heparan sulfate proteoglycans (HSPGs) are complex macromolecules commonly found on cell surfaces and within the extracellular matrix (ECM). They consist of a core protein to which long chains of heparan sulfate (HS), a linear polysaccharide, are covalently attached. These chains comprise repeating disaccharide units composed of either glucuronic acid or iduronic acid linked to N-acetylglucosamine. Due to the heavy sulfation pattern, HS is the most negatively charged biopolymer in nature. This feature allows HSPGs to interact with diverse biomolecules in the ECM or on the cell surface in various biological processes, fulfilling numerous essential roles. In the ECM, HSPGs provide mechanical stability and structural integrity by interacting with other components such as fibronectin, laminin, and collagen. They also serve as adhesion sites for cells, facilitating cell migration and adhesion while enabling cell signaling and communication through interactions with cell surface receptors like integrins or secreted signaling molecules. Extracellular HSPGs are also crucial for the formation and maintenance of basement membranes and other cellular barriers, regulating the selective passage of molecules and cells between different tissue compartments, whereas the cell surface HSPGs are involved in regulating the activities of growth factors, cytokines, and chemokines by acting as co-receptors, reservoirs, or modulators.

In addition to their physiological roles, HSPGs have been implicated in the pathogenesis of various diseases, including cancer (32), inflammation, and infectious diseases. Altered expression and function of HSPGs have been observed in various cancers, which is thought to regulate tumor growth, angiogenesis, invasion, and metastasis. In inflammatory diseases, HSPGs participate in processes by facilitating the recruitment of immune cells to the site of inflammation and cell adhesion and migration. Additionally, HSPGs can regulate immune response directly (33).

**Figure 2:**
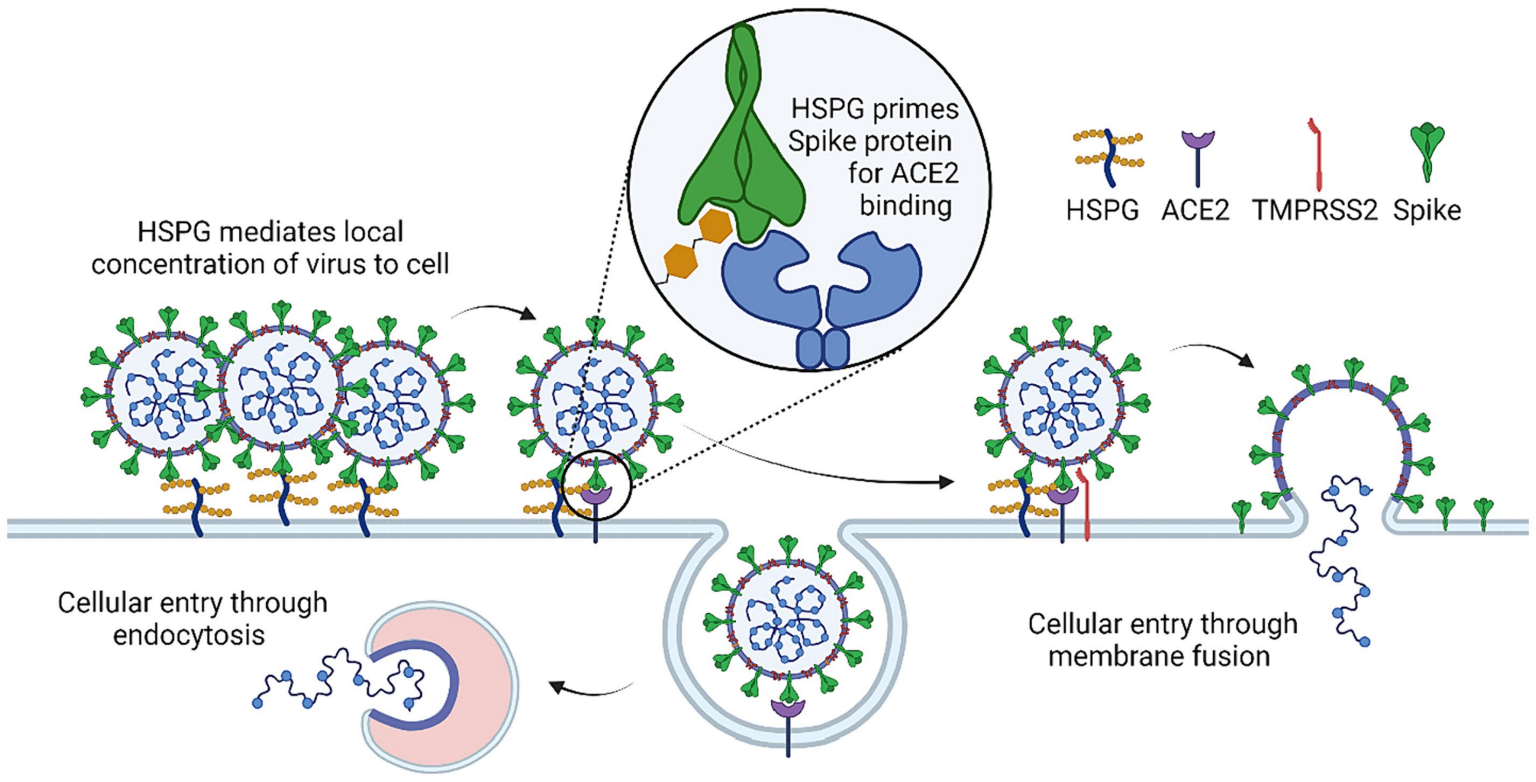
Mechanism of HSPG co-factor in SARS-CoV-2 infection. HSPG initially causes accumulation of SARS-CoV-2 at the cell surface due to binding of SARS-COV-2 spike and HSPG. HSPG binding triggers a conformational change that leads to the “open” confirmation necessary for spike-ACE2 binding. Spike-ACE2 binding will either lead to cellular entry through endocytosis or cleavage of spike at the cell surface by TMRPSS2 which leads to cellular entry through direct membrane fusion.

In infectious diseases, HSPGs can often serve as a recruiting factor to facilitate viral attachment to the cell surface. Indeed, recent studies suggested that the interaction between the Spike protein and ACE2 alone is not sufficient for efficient entry of SARS-CoV-2. Instead, the virus appears to use HSPGs as a co-receptor (3, 34–36), which uses the HS polysaccharide chains to interact with the spike protein primarily through the receptor-binding domain (RBD). This interaction has been shown to be essential for viral attachment and entry, particularly in cells that express low levels of ACE2 (35, 37, 38) (Figure 2).

In addition to enhancing viral attachment to cells, HSPGs may also regulate the conformational change of the spike during viral entry. The spike protein contains 22 N-linked glycans per protomer, which form a glycan shield around much of the protein and help to mask antigenic sites for immune evasion (39). These glycans may also help to drive a conformational change in favor of an open state in the spike protein, a process that may involve HSPGs. Multiple studies have shown that the predicted HS binding site on RBD overlaps significantly with the RBD binding site for N-glycan at N165, which suggests that HSPGs may stabilize RBD in the open conformation (39–43).

Interestingly, HSPG has been shown to form a ternary complex with ACE2 and spike, promoting SARS-CoV-2 endocytosis (3, 35, 44). Accordingly, drugs that bind to HS and HS mimetic compounds have been found to inhibit endocytosis-mediated entry of SARS-CoV-2 (3, 41, 43, 45–47). Additionally, for SARS-CoV-2 variants that enter cells via the cell surface, a recent study reveals an unexpected function of HS in regulating infection-associated cell–cell fusion, a process generating large multi-nuclear syncytia in association with severe COVID-19 cases. Specifically, HS promotes the clustering of ACE2 on the cell surface, which concentrates the spike protein on neighboring cells to maintain their fusogenic activity. The SARS-CoV-2 fusogenic activity may link to the transmissibility and symptom severity of variants. A HS-binding small molecule was shown to inhibit ACE2 clustering and thus the formation of fusion pore (4). These findings suggest that targeting HSPGs may be a promising therapeutic approach for the treatment of COVID-19.

## Involvement of HS in viral entry into host cells in other viruses

In addition to their role in SARS-CoV-2 viral infection, HSPGs are also involved in other viral infections. For human immunodeficiency virus (HIV), the virus infects host cells by binding to CD4 and a chemokine receptor. HSPGs interact with HIV envelope glycoprotein gp120 through its heparan sulfate chain. This electrostatic interaction helps to concentrate the virus on the cell surface and thus enhances its affinity to CD4 and the co-receptor CCR5 and CXCR4 (48). Accordingly, soluble heparin or heparan sulfate can competitively inhibit viral binding to the cell surface (49).

In the case of herpes simplex virus (HSV), the viral glycoproteins, particularly gB and gC, can also interact with HS chains on the host cell surface, which likewise facilitates the subsequent binding of the virus to its specific entry receptors, such as nectin-1 or herpesvirus entry mediator (HVEM), and viral entry via membrane fusion (50).

Human papillomavirus (HPV) is a non-enveloped virus with a capsid composed of two structural proteins L1 and L2. The virus takes advantage of the interaction between the viral capsid protein L1 and HSPGs, which promotes a series of conformational changes in the viral particle. This process leads to the subsequent binding of the viral particle to specific entry receptors and internalization via endocytosis (51). A high-resolution crystal structure of the HPV16 L1 pentamer showed that the binding site for heparan sulfate is located on the surface of the L1 protein. This binding site consists of a highly conserved, positively charged region that interacts with the negatively charged HS chains of HSPGs (52).

Dengue virus (DENV), a member of the Flavivirus genus, is responsible for causing dengue fever, a significant public health concern in tropical and subtropical regions. Like other viruses, DENV relies on host cell surface molecules to facilitate entry and initiate infection. The interaction between DENV and HSPGs occurs via the viral envelope (E) protein, which again binds to negatively charged HS chains on HSPGs. Pretreatment of cells with heparinase, an enzyme that cleaves HS chains from HSPGs, significantly reduced DENV infectivity (28). After the initial attachment to HSPGs, DENV undergoes receptor-mediated endocytosis, which is triggered by the interaction between the virus and additional cellular receptors (53).

Zika virus (ZIKV), is also a member of the Flavivirus genus, and is closely related to dengue virus (DENV). Zika viral infection is associated with severe neurological complications, such as microcephaly in newborns and Guillain-Barré syndrome in adults. Like in the dengue infection, HSPGs have been implicated in the attachment and entry of ZIKV into host cells, which is competitively inhibited by heparin, a soluble HS mimic (54). Overall, HSPGs act as co-factors for the infection of a diverse array of RNA and DNA viruses and thus present an enticing target for pan-active antiviral discovery.

## Therapeutic development for SARS-CoV-2

Pharmaceutical companies and researchers worldwide have been working relentlessly to develop effective antiviral therapeutics to treat and prevent COVID-19. While several antiviral drugs have approvals from the US Food and Drug Administration (FDA), it is crucial to continue improving existing treatments and develop novel therapies to combat the ever-evolving virus. Here we summarize some recent developments (Table 1).

**Table 1:** Pros, and cons of the current therapeutics developed for SARS-CoV-2.

Treatment category	Pros	Cons	Clinical trial information
Replication Inhibitors	Directly target viral replication mechanisms, potentially reducing viral load. Can be effective early in infection.	Risk of developing viral resistance. May have limited efficacy if used late in the disease course.	Numerous trials for agents like Remdesivir (NCT04280705) and Molnupiravir.
Antibodies	Provide immediate immunity. Specific against SARS-CoV-2 spike protein, blocking virus entry into cells.	High cost and accessibility issues. May be less effective against new variants if they evade existing antibody specificity.	Trials for monoclonal antibodies such as Bamlanivimab and Etesevimab (NCT04427501).
Vaccines	Induce long-term immunity. Reduce severity of disease and transmission rates.	Time required to develop, test, and distribute. Variants may reduce vaccine efficacy.	Pfizer-BioNTech (NCT04368728), Moderna (NCT04470427), and AstraZeneca (NCT04516746) vaccines.
Immune Modulators	Target the host’s immune response to reduce severity of symptoms. Can be used in severe cases to prevent or treat cytokine storm.	Potential for immunosuppression, increasing risk of secondary infections. Effects can vary widely between individuals.	Trials for corticosteroids like dexamethasone (RECOVERY Trial) and IL-6 inhibitors.

Pros and Cons of replication inhibitors, antibodies, vaccines, and immune modulators on SARS-CoV-2 infection treatments have been summarized along with clinical trial information.

## Replication inhibitors

Remdesivir, a broad-spectrum antiviral drug initially developed for Ebola, was the first drug to receive FDA approval for the treatment of COVID-19. It works by inhibiting the viral RNA-dependent RNA polymerase, thus disrupting viral replication. However, its clinical application is limited due to poor efficacy, particularly in patients with severe disease (55). Molnupiravir, a drug developed by Merck and Ridgeback biotherapeutics, is an oral antiviral drug that also inhibits the viral RNA polymerase. It introduces errors into the viral RNA genome during replication, eventually leading to the collapse of the viral population. It reduces the risk of hospitalization and death of high-risk patients with mild to moderate symptoms. However, its safety and efficacy in pregnant women and immunocompromised patients are unclear that raised safety concerns (56). Paxlovid, developed by Pfizer, is an oral antiviral drug that has also received Emergency Use Authorization (EUA) from the FDA for the treatment of mild to moderate COVID-19 in high-risk patients. The medication consists of a combination of nirmatrelvir, a SARS-CoV-2 protease inhibitor with a short half-life *in vivo*, and ritonavir, a drug that increases the plasma concentration of nirmatrelvir by slowing its metabolism. Paxlovid prevents the virus from replicating by inhibiting the activity of the viral 3CL protease. Clinical trials have demonstrated that early administration of Paxlovid significantly reduces the risk of hospitalization and death in COVID-19 patients. However, Paxlovid has severe drug–drug interaction side effects due to potent inhibition of cytochrome P4503A4 (CYP3A4) by ritonavir, making it unsuitable for patients with underlying diseases (57–59).

## Antibody therapy

With the urgent need for effective treatments at the early phase of pandemic, antibody therapy has gained tremendous attention due to its safety profile and the potential for neutralizing the virus and modulating the immune response. Antibody therapy can be achieved by using either convalescent plasma, monoclonal antibodies (mAbs), or engineered antibodies such as nanobodies. Convalescent plasma therapy involves the transfusion of plasma from recovered COVID-19 patients to individuals with active infection. This plasma contains neutralizing antibodies against SARS-CoV-2, which can potentially block viral entry and eliminate the virus. While initial studies showed some hope, convalescent plasma therapy has only demonstrated limited success due to variable efficacy and safety concerns due to the risk of transfusion-related complications. Monoclonal antibodies (mAbs) are laboratory-produced molecules engineered to target specific viral proteins. Several mAbs targeting the SARS-CoV-2 spike protein, such as bamlanivimab, etesevimab, casirivimab, and imdevimab, have received EUA from the FDA for the treatment of mild to moderate COVID-19. Clinical trials have demonstrated reduced viral load and decreased hospitalization rates in treated patients, making mAbs the primary therapeutic tool in COVID-19 therapy during the early phase of the pandemic.

However, the application of antibodies faces several limitations and challenges. The emergence of SARS-CoV-2 variants with mutations in the spike protein has reduced the efficacy of antibody therapy, and some mAbs no longer effectively neutralized novel variants and have had their approval withdrawn. Additionally, monoclonal antibody therapy requires intravenous administration, which limits its accessibility. Moreover, the high cost and limited availability of mAbs can further hinder the access to this treatment, particularly in low-income countries (60–64).

## Vaccines

The COVID-19 vaccine development efforts have employed various vaccine platforms, including mRNA, viral vector, protein subunit, and inactivated or attenuated virus. The Pfizer-BioNTech and Moderna vaccines are mRNA-based, while the AstraZeneca, Johnson & Johnson, and Sputnik V vaccines use viral vector platforms. Sinovac, Sinopharm, and Bharat Biotech have developed inactivated virus vaccines. Protein subunit vaccines, such as the one developed by Novavax, are now also entering the market. The mRNA vaccines have demonstrated high efficacy in clinical trials, with the Pfizer-BioNTech vaccine showing 95% efficacy and the Moderna vaccine showing 94% efficacy reduced symptomatic cases. The viral vector vaccines have also demonstrated high efficacy, with the AstraZeneca vaccine showing 76% efficacy after two doses and the Johnson & Johnson vaccine showing 72% efficacy in the United States. The inactivated virus vaccines have shown lower efficacy, with Sinovac showing 50.4% efficacy in a Brazilian trial and Bharat Biotech showing 78% efficacy in an Indian trial. One of the critical challenges in the COVID-19 vaccine rollout was to ensure equitable access to vaccines. High-income countries have secured a large proportion of the available vaccine doses, while low-and middle-income countries have struggled to obtain sufficient supplies. Efforts were taken to address this disparity through initiatives such as the COVAX program, which aims to provide vaccines to low-and middle-income countries (65–68).

The global effort has led to the development and distribution of COVID-19 vaccines at a speed unseeable in the human history, highlighting the power of scientific collaboration and innovation. The rapid rollout of effective COVID-19 vaccines has proven to be a critical factor in the fight against the pandemic, but logistical challenges (e.g., continuing emergence of new virus variants), particularly for mRNA-based vaccines, remain unresolved.

## Other therapeutic options for patients with severe symptoms

While most patients experience mild to moderate symptoms, a subset of individuals develop severe illness due to strong inflammatory responses, which may result in acute respiratory distress syndrome (ARDS), multi-organ failure, and death.

Immune modulators targeting specific components of the immune system have been used to reduce inflammation and prevent severe complications in COVID-19 patients. Two commonly used immune modulators for COVID-19 patients are tocilizumab and baricitinib. Tocilizumab is a monoclonal antibody that blocks the action of interleukin-6 (IL-6), which plays a key role in the body’s immune response. Elevated levels of IL-6 have been observed in some COVID-19 patients, particularly those with severe disease, and have been associated with poor outcomes. By blocking IL-6, tocilizumab can reduce inflammation and prevent damage to organs such as the lung, liver, and kidney (69). Baricitinib is a Janus kinase (JAK) inhibitor that blocks the activity of certain enzymes involved in the immune response. It has been shown to reduce inflammation and improve clinical outcomes in COVID-19 patients when used in combination with remdesivir (28). In addition to its anti-inflammatory properties, baricitinib may also have antiviral effects as it may help to reduce viral load in infected patients (70).

Corticosteroids have been used for decades to treat a wide range of inflammation-associated medical conditions, including autoimmune diseases and cancer. In the context of COVID-19, corticosteroids have been used to reduce inflammation in the lung and prevent the immune system from overreacting to the virus. However, the use of corticosteroids in COVID-19 patients has been controversial, with some studies suggesting beneficial effect while others raising concerns about their potential risks (71). Several randomized controlled trials have investigated the use of corticosteroids in COVID-19 patients, including the RECOVERY trial, which included over 6,000 hospitalized COVID-19 patients across the United Kingdom. The trial found that the use of dexamethasone, a commonly used corticosteroid, reduced mortality in COVID-19 patients who required supplemental oxygen or mechanical ventilation. Similar findings have been reported in other trials (72, 73). However, corticosteroids can also have adverse effects, particularly when used in high doses or for prolonged periods. These side effects can include increased risk of infections, diabetes, and psychiatric disturbances (74).

## Therapeutic development based on the structure of HSPGs in COVID-19 infection

Given the critical role played by HSPGs in SARS-CoV-2 infection, several studies have explored the possibility of targeting the cell surface HS as a new therapeutic strategy. Heparin, a widely used anticoagulant, has been explored for its potential benefits in treating COVID-19 patients. One study found that heparin treatment, particularly in patients with severe COVID-19 and coagulopathy, was associated with better prognosis and reduced mortality (75). However, the efficacy of heparin on patients appear to vary from case to case, depend on the disease severity: In noncritical illness, therapeutic dose of anticoagulation with heparin increased the probability of survival and hospital discharge with reduced cardiovascular or respiratory organ support. However, in critical illness situation, heparin treatment does not render significant benefits (76, 77). Highly sulfated synthetic and semi-synthetic glycosaminoglycans of different structures, known as heparan sulfate mimetics (HSMs), have been shown to have antiviral effects in *in vitro* studies by different laboratories (40, 78–80). Several heparin-like drugs commonly used to treat other diseases have also been found to have anti-SARS-CoV-2 activity. For example, Pixatimod, a synthetic HS mimic currently in clinical trials for cancer, was found to disrupt the interaction between the spike protein and ACE2, thereby inhibit SARS-CoV-2 infection (45). Pentosan polysulfate, a semi-synthetic heparin-like glucosaminoglycan used to treat interstitial cystitis, exhibits weaker anticoagulant effects but a more potent SARS-CoV-2 inhibitory activity than heparin in Vero cells *in vitro* model (81). Mucopolysaccharide polysulfate, a heparinoid used clinically for its antithrombotic effect, also has an antiviral activities against wild-type and Delta SARS-CoV-2 in an *in vitro* cell-based assay, which appears more effective than heparin (82). Sulodexide, a mixture of fast-moving heparin and dermatan sulfate, used clinically to prevent and treat vascular diseases, were shown to improve the clinical outcomes of COVID-19 patients (83).

Marine-derived sulfated glycans, with their structural resemblance to heparan sulfate, emerge as viable mimetics capable of thwarting viral attachment and subsequent cell entry. Investigations into these compounds have illuminated their broad-spectrum antiviral capabilities, underscored by studies that suggest their ability to inhibit viral entry (80, 84–87). Mechanistic studies further elucidate the specific interactions by which these glycans obstruct viral binding to HSPGs, providing a foundation for therapeutic optimization (88, 89). Critical to the advancement of these compounds are evaluations of their safety profiles and pharmacokinetic properties, pivotal for ensuring their viability as therapeutic options in clinical trials. Collectively, these studies underscore the potential of marine-based sulfated glycans as innovative anti-SARS-CoV-2 therapeutics, highlighting the necessity for continued research to fully harness their therapeutic promise.

Along a similar direction, we recently discovered a potent HS binding drug Mitoxantrone from a drug repurposing screen (3, 4, 47). Mitoxantrone was initially discovered as a DNA topoisomerase inhibitor and was approved by the FDA to treat acute myeloid leukemia in adults, advanced prostate cancer, and certain forms of sclerosis (90, 91). *In vitro* binding studies show that the HS binding activity of Mitoxantrone is uncoupled from its DNA topoisomerase binding activity. A structure activity relationship study combined with NMR analyses have defined the chemical moiety of Mitoxantroen involved in HS binding and determined the sulfate pattern on HS recognized by Mitoxantrone. Accordingly, a Mitoxantrone derivative named Pixantrone was discovered to have similar affinity to HS, but its clinical safety profile is significantly improved. Both Mitoxantrone and Pixantrone effectively inhibit endocytosis-mediated cell entry of SARS-CoV-2 *in vitro* and also reduce infection associated syncytium formation. In a mouse model of SARS-CoV-2 infection, Pixantrone shows a modest inhibitory activity against the viral entry and replication.

While current studies support the possibility of targeting HSPSs for COVID-19 therapy, several obstacles are present for this approach. First of all, given the abundant presence of HSPGs on the surface of many cell types, it may be difficult to effectively deliver the drug in high concentration to the lung or upper respiratory tissues. This lack of specificity could also result in unintended effects on other signaling pathways or cellular processes, which may cause adverse effects. Secondly, the pharmacokinetics and *in vivo* toxicity of HSPG-targeting therapeutics are not fully understood. It is unclear how the drugs are distributed in the body, how they are metabolized, and whether or not they could cause toxicity. Lastly, the SARS-CoV-2 virus may develop resistance to HSPG-targeting therapeutics over time, though the chance is much smaller than the direct targeting drugs. As with other antiviral drugs, resistance may emerge through the selection of viral mutations that allow the virus to escape the drug’s inhibitory effects.

## Future perspectives

Most antiviral therapeutics directly target SARS-CoV-2. However, developing antiviral drugs that target host cell proteins, such as viral receptors, cell entry-associated factors, and human proteases for viral protein priming, can also be an effective approach. These drugs have a lower likelihood of resistance development, as viral infections generally do not cause mutations in host cell proteins. For instance, HIV patients resistant to direct viral targeting drugs like nucleoside reverse transcriptase inhibitors (NNRTIs) and protease inhibitors (PIs) may still be responsive to host-targeting drugs. Approved host-targeting drugs for HIV include maraviroc (Selzentry, a small molecule CCR5 antagonist) and ibalizumab (Trogarzo, an antibody that binds to CD4 as a post-attachment inhibitor) (92, 93).

Future drug development for COVID-19 requires innovative strategies to discover new drug targets. One approach involves developing small molecules that target other essential viral proteins, such as nonstructural proteins (nsps), which play crucial roles in viral replication and are less prone to mutation, minimizing the potential for drug resistance. Another approach is to utilize combination therapy, wherein antiviral drugs with different mechanisms of action are combined to enhance efficacy. Additionally, there is a need to develop broad-spectrum antivirals effective against multiple coronaviruses to prepare for future outbreaks and offer a more extensive range of treatment options.

**Figure 3:**
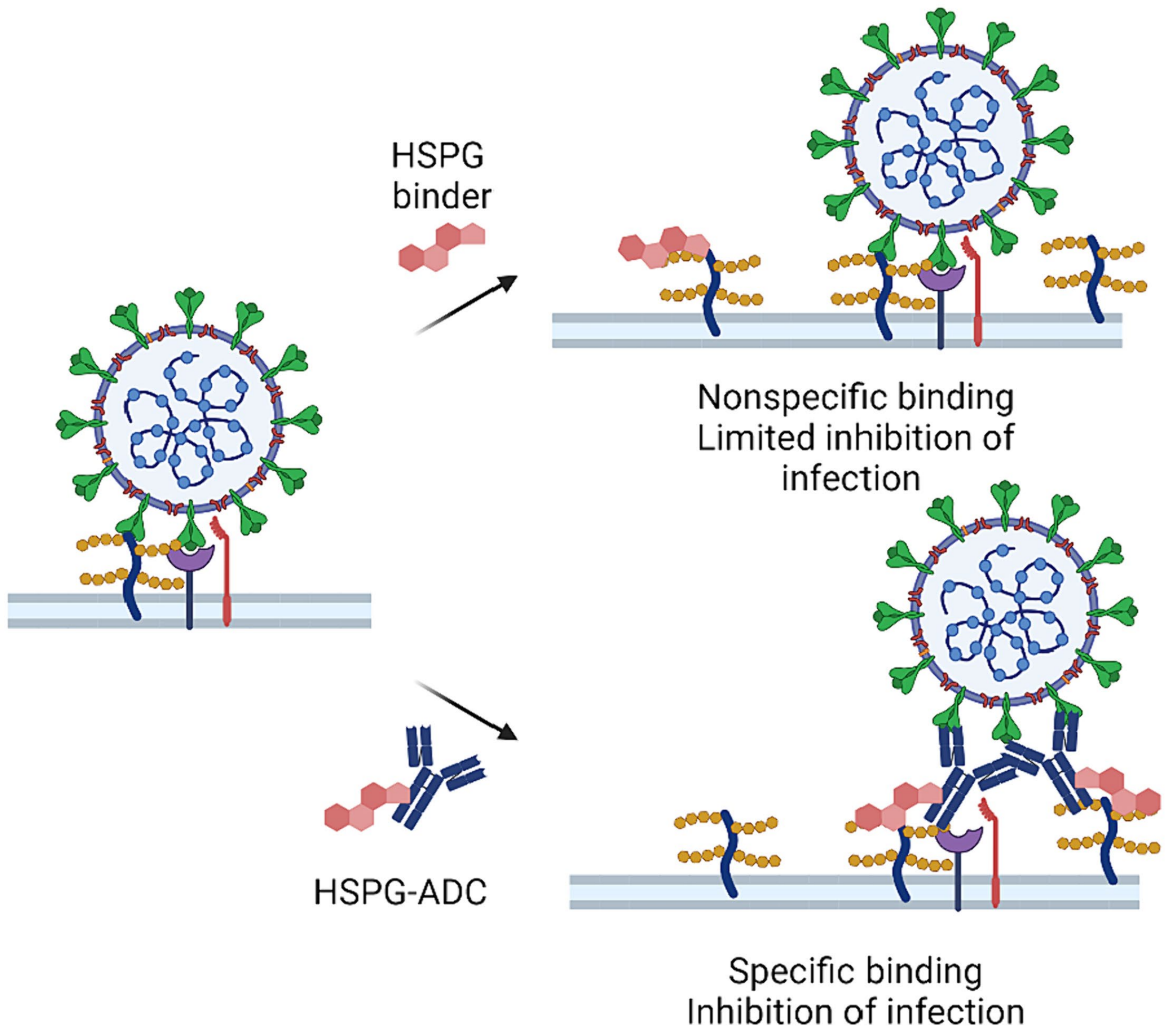
HSPG-ADC conjugates can improve inhibition of HSPG targeting of SARS-CoV-2. There is a large variety of HSPGs at the cell surface making it difficult to saturate all HSPGs that may serve as co-factors in viral infection. Conjugation of HSPG binders to antibodies that target viral surface protein or other host factors can improve efficacy by targeting HSPG-binders at the site of viral infection.

Future studies should also focus on developing therapeutics with improved specificity in target selection. Antibody-drug conjugates (ADCs) may offer a potential solution. ADCs are a class of biopharmaceuticals that use monoclonal antibodies to enhance the specificity of a conventional small-molecule drug. ADCs have shown promising effects in anti-cancer treatment, but their application in COVID-19 treatment is still in its early stages (94–96). Conceptually, the strategy of conjugating HS-targeting compounds to a spike-neutralizing antibody may present a future avenue for anti-HS-based therapeutic development. By combining the specificity of spike-neutralizing antibodies with a HS targeting drug, ADCs could potentially enhance the potency and specificity of COVID-19 therapeutics, while minimizing undesired binding to other tissues (Figure 3). Another solution is to develop inhalable HS-targeting drugs that block HS binding sites on the surface of nasal and upper respiratory tract, which can reduce the infection risk with improved tissue specificity. Inhalable HS inhibitors may also reduce lung inflammation as they can inhibit infection-associated cell–cell fusion in the lung. Further research is needed to explore these possible approaches, which may lead to clinically effective HS-targeting therapies for COVID-19 and other infectious diseases. In conclusion, directly targeting HSPG for SARS-CoV-2 is a promising approach for future drug development. The host targeting HSPG inhibitors may also have efficacy against many other viruses such as HIV, herpes simplex virus, Human papillomavirus, Zika virus, and Dengue viruses.

The therapeutic management of individuals co-infected with SARS-CoV-2 and other viruses, such as those living with HIV, necessitates a comprehensive and nuanced approach that considers the interactions between the infections and the patient’s underlying health conditions. For people living with HIV, it is paramount to continue antiretroviral therapy (ART) to maintain viral suppression and optimize immune function, alongside adhering to established COVID-19 treatment protocols that may include antivirals, immunomodulatory agents, and supportive care, all while vigilantly monitoring for potential drug–drug interactions (97–100). It is possible that targeting heparan sulfate proteoglycans (HSPGs) could offer therapeutic benefits for HIV-positive individuals co-infected with SARS-CoV-2 given the proposed role of HSPGs in facilitating HIV entry into cells (48). However, the effectiveness of HSPG-targeted therapies in such co-infected individuals may depend significantly on their immune status and the presence of any existing comorbidities. Given the complexity of these interactions and the potential for varied responses to treatment, there is a pressing need for clinical trials specifically designed to evaluate the efficacy of HSPG-targeted treatments in populations co-infected with HIV and SARS-CoV-2. This approach underscores the importance of a personalized medicine strategy that considers each patient’s unique clinical profile. Ultimately, advancing our understanding of the most effective therapeutic strategies for co-infected individuals will require ongoing research and the integration of findings from across the spectrum of virology, immunology, and pharmacology.

## Author contributions

QZ: Writing – original draft, Writing – review & editing. IP: Writing – original draft, Writing – review & editing. YY: Writing – original draft, Writing – review & editing. WZ: Funding acquisition, Resources, Writing – original draft, Writing – review & editing.
